# Multitalented Synthetic Antimicrobial Peptides and Their Antibacterial, Antifungal and Antiviral Mechanisms

**DOI:** 10.3390/ijms23010545

**Published:** 2022-01-04

**Authors:** Tania Vanzolini, Michela Bruschi, Andrea C. Rinaldi, Mauro Magnani, Alessandra Fraternale

**Affiliations:** 1 Department of Biomolecular Sciences, University of Urbino Carlo Bo, 61029 Urbino, PU, Italy; t.vanzolini@campus.uniurb.it (T.V.); mauro.magnani@uniurb.it (M.M.); alessandra.fraternale@uniurb.it (A.F.); 2 Department of Biomedical Sciences, University of Cagliari, 09042 Monserrato, CA, Italy; rinaldi@unica.it

**Keywords:** antimicrobial peptides, antifungal, antibacterial, antiviral, peptide-based therapies, synthetic peptides

## Abstract

Despite the great strides in healthcare during the last century, some challenges still remained unanswered. The development of multi-drug resistant bacteria, the alarming growth of fungal infections, the emerging/re-emerging of viral diseases are yet a worldwide threat. Since the discovery of natural antimicrobial peptides able to broadly hit several pathogens, peptide-based therapeutics have been under the lenses of the researchers. This review aims to focus on synthetic peptides and elucidate their multifaceted mechanisms of action as antiviral, antibacterial and antifungal agents. Antimicrobial peptides generally affect highly preserved structures, e.g., the phospholipid membrane via pore formation or other constitutive targets like peptidoglycans in Gram-negative and Gram-positive bacteria, and glucan in the fungal cell wall. Additionally, some peptides are particularly active on biofilm destabilizing the microbial communities. They can also act intracellularly, e.g., on protein biosynthesis or DNA replication. Their intracellular properties are extended upon viral infection since peptides can influence several steps along the virus life cycle starting from viral receptor-cell interaction to the budding. Besides their mode of action, improvements in manufacturing to increase their half-life and performances are also taken into consideration together with advantages and impairments in the clinical usage. Thus far, the progress of new synthetic peptide-based approaches is making them a promising tool to counteract emerging infections.

## 1. Introduction

When Fleming in 1922 discovered the first natural antibiotic, the lysozyme, [1] able to “lyse” bacterial cells and in 1928, Penicillin, from the fungus *Penicillium notatum*, able to inhibit bacterial growth, [2] the dawn of the antibiotic age started. Later on, in 1939, René Dubos isolated an antibacterial agent from *Bacillus brevi*, called gramicidin [3]. Gramicidin demonstrated its broad-spectrum activity against Gram-positive and Gram-negative bacteria becoming the first antibiotic commercially manufactured and sold up to this day [4]. Since the discovery of human defensins, histatins and cathelicidins, antimicrobial peptides (AMPs) have been studied, sequenced, and synthesized in laboratory in order to be used in the clinic for the treatment of several bacterial, fungal and viral infections. Besides representing the first defense of the innate immune system against pathogens, [5] they also have immunomodulatory effects working as mediators of the infection-associated inflammation, recruiting, and enhancing the activity of leukocytes and the release of cytokines but also contributing to the infection control and resolution [6,7].

Besides humans, natural AMPs have been found in different kingdoms (animals, plants, bacteria, fungi but also archaea and protists) and registered in the AMP database (https://aps.unmc.edu/, accessed on 3rd November 2021) [8,9]. Briefly, all AMPs share common features, such as a sequence composed of less than 100 amino acids (aa), [10] with the majority having between 10 and 60 aa [11]. Even if some anionic AMPs, rich in glutamic and aspartic acids, are negatively charged [12], almost all antimicrobial peptides have a net positive charge for the presence of a high number of lysine, arginine and histidine (protonated in acidic conditions) [13]. Finally, another common feature is represented by the hydrophobicity conferred by hydrophobic aa that often overcomes 50% of the total amino acid sequence [14]. The high lipophilicity is useful especially for the penetration in the biological membranes but considering the net charge, overall, AMPs are amphipathic molecules. The classifications are based on their structure or the presence/absence of recognizable motifs. AMPs could be α-helix, β-sheet, linearly extended, both α-helix and β-sheet, cyclic and with complex structure or, seen from a different perspective, tryptophan- and arginine-rich, histidine-rich, proline-rich and glycine-rich [15,16].

In the last decades, the increasing resistance to antibiotic treatments, i.e., Methicillin, Vancomycin-resistant *Staphilococus aureus* and the rise of species with intrinsic multi-drug resistance, such as *Candida auris*, highlights the need for the development of new agents [17,18,19]. It has been estimated that nowadays in the US every year 2.8 million people are infected by antibiotic-resistant microorganisms with a death rate of 35,000 people [20] and just in recent years the world was affected by a new pandemic virus (SARS-CoV-2) with 236 million cases and 5.9 million deaths up-to-date [21].

Studies on the AMPs synthetic analogs provided a new tool to understand the different and unique modes of actions against diverse microorganisms. Thus, this review will focus on the improvements of their properties with respect to their natural counterpart, their activity on bacterial and fungal conserved structures, i.e., membranes and cell walls, as well as on biofilm formation, their antiviral properties and execution dynamics.

The latest studies in vivo and in vitro will be discussed, with highlights on the successful therapeutic application despite drawbacks like toxicity and immunogenicity.

## 2. Synthetic Antimicrobial Peptides

Natural antimicrobial peptides have been always present during the evolutionary process [22], however, many natural AMPs showed host toxicity, rapid degradation by proteases, instability due to pH changes, loss of activity in presence of serum and high salt concentrations, lack of suitable delivery systems able to limit the drawbacks, and high costs of production [23,24,25]. Moreover, their complex design, low antimicrobial activity and pharmacokinetics led many laboratories to improve their structure and amino acid sequence to enhance their therapeutic properties [26]. Despite the multiple obstacles in the clinical application, synthetic peptides were developed to overcome the difficulties linked to the natural peptides while mimicking their pharmacological qualities [27].

The approaches commonly used for the development of non-natural AMPs are (1) the site-directed mutations characterized by the addition, the deletion or the substitution of aa, (2) the de novo design which doesn’t use any template sequence, (3) the template-based design that uses fragments of the parental compound as starting point for the construction of new AMPs (in this case, antibodies seem to be a big source of patterns, especially those which recognize and bind components of the cell membrane and wall), and lastly (4) the self-assembly-based design that exploits the formation of simple nanostructures like dimers, or more complex as micelles, vesicles and nanotubes [11].

Semi-synthetic AMPs maintained the active sites of the natural source, but chemical changes were brought in order to reach the optimal properties whereas synthetic AMPs are obtained from chemical synthesis with frequent usage of the solid phase. This technique is based on the addition of one aa at a time, thus favoring the investigation of the role of each amino acid in the sequence [28].

Apart from the solid-phase method, synthetic AMPs can also derive from the catalytic ring-opening polymerization (ROP) of α-amino acid *N*-carboxyanhydride (NCA), an exquisite tool for the fabrication of long polypeptides with low polydispersity but variable chemical composition and topology [29]. Chemical synthesis represents a great step forward in peptide production with higher efficiency, reliability, and speed, especially when compared to the AMPs produced through the technology of the recombinant DNA followed by bacterial expression and purification.

The advances in the AMPs synthesis are the result of several studies about machine learning and algorithms able to predict or identify potential sequences based on the physicochemical and structural properties and on the quantitative structure-activity relationship (QSAR) of AMPs and targets already present in databases followed by high-throughput screenings [30]. Therefore, several strategies were tested to achieve a superior half-life e.g., the usage of D-amino acids [31], peptide cyclization [32], unnatural amino acids [33]. With peptidases able to recognize mainly L-amino acids sequences, stereogenic D-variants of amphipathic peptides could be resistant to proteolysis [34], as well as peptides with uncommon amino acids, i.e., ω and β-amino-acids [35,36]. Protection from cleavage could be also conferred by modifying or protecting vulnerable peptide bonds so that they cannot be easily accessed [37]. In some cases, such modifications could be applied just to the *N*- and *C*-terminus i.e., *C*-amidation or *N*-acetylation [38].

Similarly, PEGylation, the covalent attachment of polyethylene glycol (PEG) chains to lysine or to the N-terminus [39], could also be applied to mask other residues like arginine [40]. On the other hand, lipidation, consisting in the attachment of one or more fatty acid chains to a lysine residue or to the amine of the N-terminus, [41] could improve AMPs properties by enhancing their interaction with the membranes. Introduction of sulfonamide groups has been also investigated to exploit their bio-active properties, enhance their proteolytic stability and hydrogen bonding ability [42].

**Figure 1:**
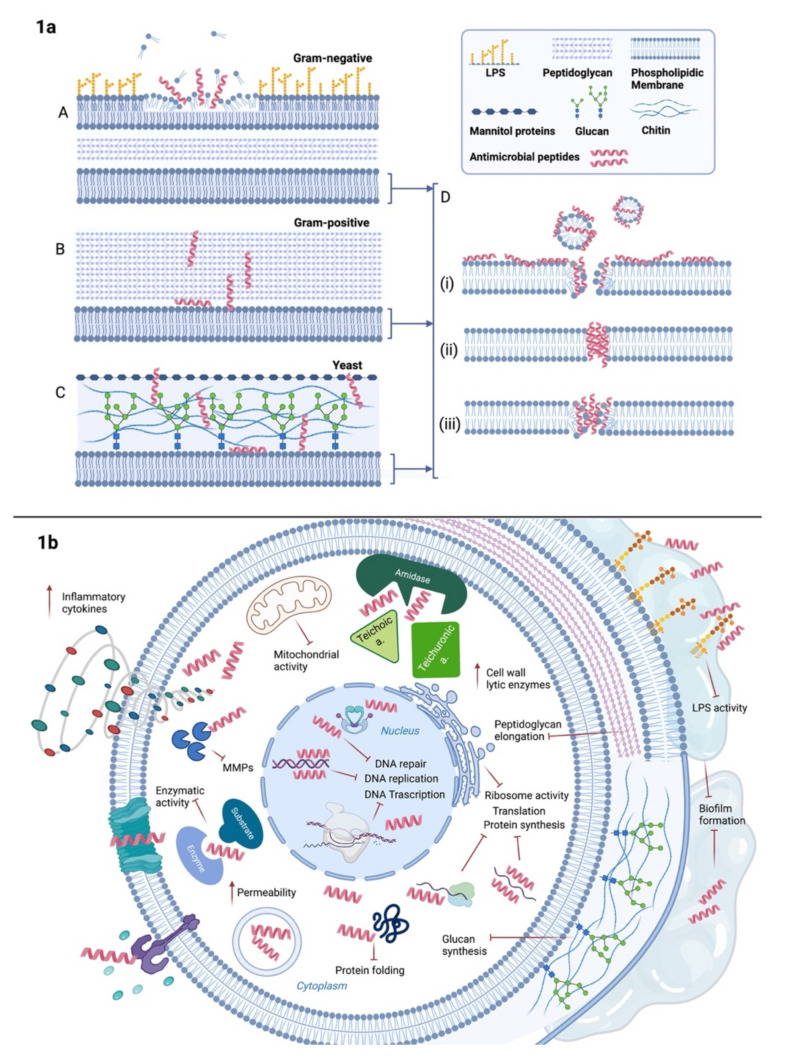
AMPs broad-spectrum antimicrobial activity. (**a**) Primarily, AMPs’action is based on their action on cytoplasmic membranes, i.e., perturbation or disruption. However, in presence of Gram-negative bacteria (**A**) AMPs have to firstly cross the outer phospholipidic membrane and secondly traverse the peptidoglycan layer before reaching the inner membrane. In Gram-positive bacteria (**B**) they navigate through the thick cell wall of peptidoglycan and in fungi (**C**), they encounter mannitol proteins, glucans and chitin prior to access to the cytoplasmic membrane. Once reached the phospholipidic bilayer, they induce perturbation via pore formation following either (**D**) (i) *carpet-like*, (ii) *barrel-stave* or (iii) or *toroidal pore* model depending on the peptide composition. (**b**) Besides pore formation, some AMPs bind some components and receptors on the extracellular side of the membrane, i.e., Toll-like receptors; others manage to enter the cytosol through direct penetration in vesicles or channels thus destabilizing the permeability and activating the inflammatory cytokines cascade. Intracellularly, they could also interfere with DNA or RNA leading to degradation and cell death. They may also affect mitochondrial activity or protein synthesis by targeting ribosome subunits or protein folding. In the case of bacterial cell wall, they can prevent elongation of peptidoglycan chains or hinder teichoic and teichuronic binding acids to amidases. Cell wall components inhibition will promote cell autolysis. In the extracellular space, AMPs can sequestrate LPS reducing the impact of endotoxins on the host’s immune response. In fungal cells, AMPs can intervene on glucan synthesis thus blocking the building pieces of their wall. Further inhibitory action on biofilm matrix impairs the *quorum sensing* and improves the susceptibility of the single pathogens in both bacterial and fungal communities.

Another approach to improve the half-life of peptides in vivo is to synthesize them as dendrimers around a residue or a linear polymer core [43]. These multiple antigen peptides (MAP) developed by Tam and colleagues [44] are mainly constituted by a lysine core to which peptide chains are attached [45]. The number of bi-, tri-, tetra and more sequence patterns define the multivalency of those peptides and confers an increased cationic charge as well as hydrophobic groups. The steric hindrance given by the bulk, firstly, limit the access to the proteolytic site [46,47] and, secondly, seems to improve their activity by increasing the local concentration of peptide units with membranolytic activity [48]. Peptide structure is a pivotal point for the interaction with the membranes: the cationic charge allows the initial binding to a negatively charged layer; afterwards, while amphipathicity is necessary for membrane perturbation and peptide uptake, the hydrophobic groups are responsible for the carving [49]. Studies on the mechanism of action would divide the AMPs in two categories: *membrane disruptive* [50,51] and *non-membrane disruptive* (activity on other targets) (Figure 1) [52,53].

## 3. Antibacterial Peptides and Their Mechanism of Action

Many factors can influence membrane perturbation and disruption by AMPs, i.e., amino acids sequence, the lipid composition of the membrane, peptide concentration as well as differences in membrane composition between eukaryotic and bacterial cells allow the AMPs to distinguish a microbial target from the host. Bacterial membranes are negatively charged due to the presence of anionic phospholipids groups, e.g., phosphatidylglycerol, phosphatidylserine, while eukaryotic cells possess groups with a neutral charge, e.g., phosphatidylcholine and phosphatidylethanolamine [54]. Moreover, the presence of cholesterol, a common feature in eukaryotic cells, is able to interact with AMPs either neutralizing or reducing their activity or stabilizing the phospholipid bilayer [55].

In Gram-positive bacteria, AMPs have to cross first the cell wall composed of crosslinked peptidoglycan with lipoteichoic acid prior to reaching the membrane whereas in Gram-negative they face a coat of lipopolysaccharide (LPS) followed by a phospholipidic outer membrane and a less cross-linked peptidoglycan layer [56]. Electrostatic interactions between the cationic peptide and the negatively charged components, e.g., lipopolysaccharide in Gram-negative and teichoic acid in Gram-positive, are the first steps to contribute to bacterial membrane affinity [57]. However, while AMPs seem to traverse the peptidoglycan layer with ease and access to the cytoplasmic membrane of the Gram-positive, they need to disrupt or perturb both outer and cytoplasmic membrane in Gram-negatives. Impedance in crossing or permeabilization results in loss of antimicrobial activity (Figure 1a (A,B)) [58].

In order to explain the perturbation of the phospholipidic membranes operated by the AMPs, three main models have been proposed: *carpet-like*, *barrel-stave* and *toroidal pore* (Figure 1a (D)). Generally, when the ratio of peptide/lipids is low, AMPs interact with the phospholipidic layer of the membrane in a parallel manner, defined as *carpet-like* model, and interaction among the peptides or penetration in the hydrophobic core of the bilayer are not taking place [59]. Membrane integrity is disrupted and micelles are formed as in a detergent-like process [60]. With increasing AMPs ratio, they move to a perpendicular orientation until reaching such a concentration that they can cross the membrane forming pores (1:50–1:500 and more) [61,62]. A minimum length of ~22 amino acid for α-helix peptides is required to span the phospholipid layer, while β-sheet structures necessitate a minimum of 8 [63].

In the *barrel-stave*, interaction among peptides is a prerequisite as they mimic a transmembrane pore, whereas, in the case of the *toroidal* model, peptides are loosely arranged [64,65]. Despite the perturbation of the membrane seems to vary depending on the peptides, actually, the mechanisms of action are not completely well-defined and they are partially overlapping [66]. Moreover, all these models are based on the membrane perturbation but, then, the killing effect is not always enough to provide antimicrobial activity [67].

Besides membrane disruption, recent studies showed how peptides could act on other targets as well (Figure 1b) [68]. Some AMPs have shown their efficacy by binding some components and receptors on the extracellular side of the membrane and wall, thus destabilizing the permeability and/or activating intracellular signaling pathways that have, as a response, the inhibition or the activation of several functions. An interesting example is represented by the binding of Toll-like receptors and the consequential amplification of the inflammatory response via NFkB cascade followed by activation of the immune system towards microbiological pathogens [69,70]. Other antimicrobial peptides manage to enter the cytosol through direct penetration, endocytosis (both micropinocytosis and receptor-mediated) [71], or the exploit of delivery systems [72]. There, they can affect different enzymes and intermediates involved in vital processes.

The inhibitors of the nucleic acid biosynthesis seem to have a high binding affinity for both DNA and RNA because they share with nucleic acid-binding enzymes or substrates, homologous fragments of their sequences; an interesting example is represented by DNA-binding protein histone H2A [73]. Other mechanisms use the inhibition of the enzymes involved in the DNA/RNA biosynthesis, like DNA topoisomerase I preventing DNA relaxation [74], RNA polymerase blocking the transcription [75] and gyrase impairing the supercoiling of DNA. [76] As a result, DNA/RNA degradation is induced and consequentially also cell death. There are several inhibitors of protein biosynthesis which alter the transcription and the translation but also the correct folding and the degradation of the protein. Usually, the AMPs that act on the protein biosynthesis target the ribosome subunits [77] but some others can interfere with the incorporation of histidine, uridine and thymidine [78,79], the amino acid synthesis pathways [80], the release factors on the ribosome [81], the regulation of sigma factors [82], the nucleotide and coenzyme transport [80] and the degradation of DNA-replication-associated proteins [83]. Some peptides influence protein folding, in particular, DnaK, the major Hsp70 of the chaperone pathway in *Escherichia coli*, which has been seen as an optimal target to prevent the refolding of misfolded proteins [84]. Another approach is linked to the inhibition of matrix metalloproteases, essential enzymes in microbial cell growth and homeostasis, i.e., serine protease, trypsin-like protease, elastase and chymotrypsins [85,86,87]. There are also inhibitors of cell division that block DNA replication or the mechanisms essential for the repair of DNA damages, then resulting in the block of the cell cycle, in the impairment of the chromosome separation, in the failure of septation, in the alteration of mitochondrial activity and in a substantial change in the cell morphology with clearly visible blebbing and elongation towards a filamentous shape [88,89].

Cell wall synthesis is another suitable target. Some AMPs act on lipid II by sequestrating it from the functional site [90,91] or by binding D-Ala-D-Ala residues of its precursor preventing the addition of *N*-acetylglucosamine and *N*-acetylmuramic acid in the structure, hence the peptidoglycan elongation [92]. Other peptides have shown antimicrobial activity by activating cell wall-associated lytic enzymes, for example, some AMPs binding teichoic and teichuronic acids which otherwise are linked to amidases. The release of amidase stimulates premature autolysin activity and, consequently, cell lysis.

Moreover, lipopolysaccharides (LPS) are components of the membrane as well but, when released, are also well-known endotoxins able to raise an excessive and harmful pro-inflammatory response. AMPs that bind and neutralize LPS avoid the excessive stimulation of the immune system favoring a correct and balanced infection resolution [93].

Recently, the AMPs inhibitory activity on biofilm has been reported. Biofilm, consisting of an extracellular matrix of mainly polysaccharides, provides virulence, persistence and drug resistance to the microbial community [94,95]. Anti-biofilm mechanisms, similar to the membrane-targeting ones, are also very diverse and sequence dependent. A database of biofilm-active peptides can be found online [http://www.baamps.it/, accessed on 10 November 2021]. AMPs could prevent biofilm formation by affecting cell attachment, or could act on preformed biofilm by disrupting the *quorum-sensing*, dispersing the cells within it, or affecting the expression of the related genes [96,97]. Destabilization of matrix architecture impairing secretion or interaction between the matrix polymers has been also hypothesized [98]. Another target is the stress-responder guanosine pentaphosphate [(p)ppGpp] a major player for biofilm growth and environmental stress resistance [99]. Weakening of the biofilm increases the susceptibility of the pathogen to the AMPs or to the conventional antibiotics, therefore, even a synergistic action could be appealing for clinical purposes [100].

## 4. Antifungal Peptides

The concern generated by bacterial infections goes hand in hand with that of fungal infections especially considering both the frequency and the rapidity their resistance develops and spreads and the poor arsenal of available antifungal drugs. Fungal infections become extremely threatening especially for certain categories represented by patients with a compromised immune system due to pathological conditions, such as HIV/AIDS or autoimmune diseases and to therapeutic outcomes like chemotherapy and organ transplantation [101]. Among the fungal species *Candida albicans*, *Aspergillus fumigatus*, *Cryptococcus neoformans* and *Pneumocystis jirovecii* are the main ones responsible for the majority of severe mycoses [102] with 90% of reported deaths [103]. Among the emerging and reemerging species, such as *Histoplasma capsulatum* and *Fusarium* spp., of note is *Candida auris* which is considered by the Centre for Disease Control and Prevention (CDC) as an urgent global threat for its multi-drug resistance [18,104].

The latest reports highlight also the need for efficient treatments that nowadays are based only on three major classes of antifungal drugs: azoles, echinocandins and polyenes. Of these classes, echinocandins originated from non-ribosomal AMPs synthetically optimized [101]. Fungi are eukaryotic organisms; hence, they share with mammalian cells high similarities making it difficult to identify suitable targets while minimizing the risk of adverse effects. Although toxicity is an important issue, synthetic modifications of AMPs structures have extremely improved safety leaving just a few exceptions mainly represented by erythrocyte hemolysis and nucleic acid damages [105,106,107]. As previously seen for antibacterial AMPs, peptides with antifungal activity may present improved affinity towards phospholipids of the fungal membrane (phosphatidylserine and phosphatidylethanolamine) suggesting a distinctive relation between structure and activity (Figure 1a (C)) [108].

The three models used to describe the pore generated in bacterial membranes (*carpet-like*, *barrel-stave* and *toroidal*) are applicable also for AMPs acting on fungal membranes. Interestingly amphotericin B, the major representative of the polyene class of antifungal drugs, behaves as *barrel-stave*-pore forming peptide [101]. Evidence has demonstrated the existence of AMPs acting on the fungal membrane and on its components without having always clear information about their mechanism of action. Often these peptides affect the permeability of the membrane leading to ROS accumulation, oxidative stress damages, ATP release and the activation of stress-response pathways as HOG and MAPK cascade [109,110,111]. On the other hand, just a few AMPs have been revealed to interact with membrane components like glucosylceramides and β-1,3-glucans or with enzymes involved in the production of membrane components as the inositol phosphoryl ceramide synthase which is essential for the sphingolipid biosynthesis [112,113,114]. Membrane-active peptides have good potential and a broad-spectrum that sometimes includes both bacteria and fungi, nevertheless, as some AMPs with exclusive antifungal properties exist, it is also the case of antimicrobial peptides active against the cell wall (Figure 1b). The cell wall is an external structure proper of fungi unique in its composition since rich in glucans, chitin and mannan. The development of cell wall-active-AMPs grants high levels of safety with no or minor toxicity for mammalian cells. Most of the AMPs interfere with the synthesis of the wall components, such as β-1,3-glucan synthase fundamental enzyme for the production of β-1,3-glucans hence for the maintenance of the structural integrity (echinocandins exert this mechanism of action) and chitin synthase essential for chitin production [106]. Mannan and its glyco—and proteo-conjugates are deeply involved in fungal virulence, biofilm formation and adhesion to both biotic and abiotic surfaces included. Mannan-binding peptides form ternary complexes with calcium able to disrupt the fungal structural integrity [115]. Other AMPs that have been investigated have identified in nucleic acids their targets, in particular, several peptides bind and intercalate the DNA or inhibit the enzymes involved in its synthesis and repair [74]. In certain cases, some antifungal AMPs altered consistently the cell morphology and the organelle functions (in particular mitochondria, nucleus and vacuole) and interact with intracellular proteins [116,117,118,119]. In addition to these modes of action, it is important to mention the innovative use in the fungal world of the cations hijacking strategy using an Aluminum and/or Iron chelator translocatable inside the fungal cell through the siderophore iron transporter 1 (Sit1) [120].

Worthy of remark is the antibiofilm activity of some antifungal peptides. Biofilm is a virulence factor that, similarly to bacteria, a community of fungal cells adopts to evade the immune system. Moreover, it provides protection from antifungal drugs since the extracellular matrix works as a penetration-delayer factor. The colonization of both biotic and abiotic surfaces followed by biofilm formation represents a great risk especially in nosocomial settings where the use of invasive devices is a normal practice. Biofilm is associated with high morbidity and mortality rates and the development of AMPs with antibiofilm potential is urgently needed. Several antifungal peptides have been widely characterized and, among their abilities, they managed to both inhibit the biofilm formation and eradicate mature biofilm [121,122,123,124,125]. A negative point is the lack of precise information about the mechanism that sometimes could be considered as a downstream consequence attributable to the modes of action just described.

## 5. Antiviral Peptides

**Figure 2:**
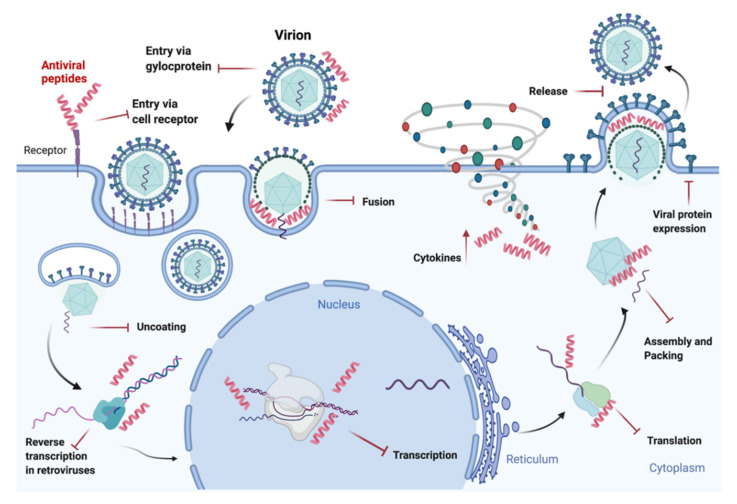
AVPs targets in viral life cycle. Depending on the type of virus and on the mode of action of the peptides, AVPs can block viral entry by binding with specific cellular receptors or interaction with viral glycoproteins, which are involved in both entry and fusion process. They may also hinder the fusion via physicochemical interaction with hydrophobic membrane–protein interfaces. AVPs can act intracellularly as well by direct influence of viral nucleic acid synthesis or blocking viral protein expression. Others modulate the antiviral immune system of the host cell by up-regulating expression of interferons and cytokines.

Viruses represent a major cause of human disease, and the emergence of viral drug resistance and epidemics induce to search for new antivirals. Natural AMPs are an interesting source of innovative antiviral agents, but more interestingly, antiviral peptides (AVPs) can be designed and optimized to block critical steps of the viral life cycle (Figure 2) [126]. In 2014, Kumar et al. described the AVP targeting about 60 medically significant viruses [127]. Usually, AVPs exhibit antiviral effects by inhibiting the virus directly, but their inhibition sites and the mechanism of action vary within the viral replication cycle.

Most viral pathogens are present in the Emerging Infectious Diseases/Pathogens list of the US National Institute of Allergy and Infectious Diseases (NIAID), such as Smallpox virus, viral hemorrhagic fever viruses (arenaviruses, bunyaviruses, flaviviruses and filoviruses), and coronaviruses are membrane-enveloped viruses. Virus and host cell membrane fusion is necessary for virus entry and biophysical as well as biochemical features of the membrane fusion process can be common among enveloped viruses. Targeting these conserved characteristics that are necessary for membrane fusion, is emerging as a new tool for the development of broad-spectrum antivirals [128].

Viral entry, which is the earliest phase of infection in the viral life cycle, is the favored target for AVPs. Most AVPs block viral entry by one of the next mechanisms: (1) interaction with heparan sulfate, (2) blocking of cell-to-cell spread, (3) interaction with specific cellular receptors, (4) interaction with viral glycoproteins, (5) membrane or viral envelope interaction [129].

Viral surface glycoproteins are involved in both the entry and penetration process and undergo conformational changes because of the interactions with the receptor proteins. Most AVP inhibit enveloped viruses’ entry by physico-chemical interaction with hydrophobic membrane–protein interfaces [130]. A few examples of peptide entry inhibitors are reported. Enfuvirtide is a peptide entry inhibitor for HIV that acts by blocking the HR1 domain of the viral envelope glycoprotein 41; it was approved by the US Food and Drug Administration (FDA) and the European Medicines Agency (EMA) for human use in 2003 [131]. Another class of HIV entry inhibitors, termed anchor inhibitors, target the fusion peptide [132].

The mimetic peptide, DN59, which consists of the amino acids corresponding to the amphipathic stem region of the dengue virus envelope glycoprotein was shown to interfere with the normal infective process [133]. Peptides homologous to the surface glycoproteins of HSV-1 and HSV-2 envelopes were demonstrated to be active against the herpes virus [134]. Peptide entry inhibitors were also used against other viruses, such as cytomegaloviruses, influenza virus and coronaviruses [130]. ACE2-derived peptides were already used to contrast SARS-CoV infection [135], and the approaches used to synthesize peptides against coronaviruses in the past may be re-considered to design new peptides for inhibition of SARS-CoV-2 infection on the documented evidence of efficacy against SARS-CoV, MERS-CoV, SARS-related CoVs. For example, among these peptides, which had already been used against SARS-CoV-1, 15 were selected against the receptor-binding domain (RBD) of the spike protein of SARS-CoV-2 potentially able to inhibit the entry of SARS-CoV-2. Moreover, peptides targeting domains in the S protein other than the RBD may also interfere with viral entry [136]. The approaches followed for the development of peptides targeting SARS-CoV-2 entry have been recently summarized by Schütz and colleagues [136].

AVPs with potential anti-SARS-CoV-2 activities could target the host as well. The mouse β-defensins-4 derived P9, thanks to its polycationic property, prevents endosomal acidification necessary for viral-host endosomal membrane fusion and consequent viral uncoating and RNA release, resulting in inhibition of the virus [137].

More recently, a dual-functional cross-linking peptide 8P9R has been demonstrated to inhibit both the endocytic pathway and the TMPRSS2-mediated pathway of SARS-CoV-2 hypothesizing its employment in effective cocktail therapy with repurposed drugs [138]. Otherwise, another target may be the ACE2 receptors instead of the viral S1 subunit [139].

Distinctively, other AVPs have been designed to modulate intracellular targets [129]. It is known that antimicrobial host defense peptides, such as PR39 and LL-37 can cross lipid membranes, while others are found as precursors inside host cell vacuoles. Cellular internalization of these peptides can stimulate gene/protein expression by blocking viral protein expression, influencing viral nucleic acid synthesis, or stimulating host cell antiviral defenses [128,140]. while others modulate the antiviral immune system of the host cell by up-regulating the expression of interferons and cytokines [141]. For example, rhesus theta-defensin 1 (RTD-1) is a cyclic antimicrobial peptide first identified in rhesus macaque leukocytes, that was demonstrated to alter pulmonary infection outcome induced by SARS-CoV in mice by potentiating cytokine responses [142,143].

Therefore, AVPs can be designed and optimized through a deep knowledge of the structures of viral proteins and cellular targets. Host cell factors proteins or pathways required by numerous viruses to complete their replication cycle are attractive targets for broad-spectrum antivirals, included AVPs. Indeed, this strategy would offer a versatile solution that could work against many viruses, including the emerging ones, offering a low possibility of inducing drug resistance. However, the major concerns duly noted are the cellular proteins function in the complex network of interactions as well as cytotoxicity.

For this reason, many peptides are designed, as described previously, to act extracellularly, i.e., to target early steps of viral replication, such as viral envelope glycoprotein activation, receptor attachment, or fusion.

Although smaller than standard AVPs, reduced glutathione (GSH) deserves to be cited as an effective antiviral against different viruses. GSH is a tripeptide, present in all mammalian cells, constituted of the amino acid L-glutamate, L-cysteine, and glycine. Its synthesis is catalyzed sequentially by γ-glutamylcysteine and GSH synthetase. Inside the cells, 98% of glutathione is found in reduced form, and only 2% is oxidized (GSSG) or joined with other molecules [144]. Glutathione (GSH) has a key role in cellular physiology and metabolism [145]. Furthermore, in the last years, an imbalance in the GSH/GSSG ratio has been described in several pathologies including viral infections [146]. It has been widely demonstrated that intracellular redox status alterations, associated with depletion of GSH are essential for the completion of the viral cycle. However, the mechanisms by which viruses induce a decrease in intracellular GSH content are different and not completely clear. Accordingly, GSH has been proposed as a potent antiviral acting with different mechanisms depending on the type of virus. Recently, the role of GSH in determining individual responsiveness to COVID-19 infection and the possibility of using GSH for the treatment and prevention of COVID-19 illness has been also described [147].

Unfortunately, GSH has a short half-life in blood plasma and hardly crosses the cell membrane; for this reason, design strategies have emerged in the development of GSH derivatives with improved permeability or small molecules able to release intracellularly precursors for GSH synthesis [148]. Many papers have reported the efficacy of GSH and pro-GSH molecules in inhibiting replication of several viruses and many reviews have summarized the results achieved over the years [146,149,150,151].

In conclusion, AVPs, due to their ability to target various aspects of the viral lifecycle, their low molecular weight and low toxicity, can be considered a potential resource to combat emerging and re-emerging viral pathogens for which drug-resistance was developed or specific therapies do not exist. Especially, in the light of recent fast-replicating viruses with high rate of mutation frequency, novel candidates with multiple mechanisms of action or synergistic effects are indeed highly desirable [152].

## 6. AMPs—Goods vs. Bads, and the Long Way towards Clinical Application

There are obvious, multiple advantages of AMPs over classical antibiotics. As previously describes, AMPs are easy to synthesize, thanks to recent advances in automated protein synthesis, or can alternatively be produced in large quantities in heterologous expression systems, either in microbial cells or in plants [153]. In addition, AMPs are largely prone to chemical modification, aimed at overcoming inherent problems, such as susceptibility to enzymatic degradation, chemical/physical instability and toxicity to host cells, thus optimizing molecules’ features and smoothing their pathway towards the clinics [154]. Broad-spectrum activity and rapid killing are other much-appreciated characteristics. Finally, AMPs are increasingly seen as a promising therapeutic alternative for treating biofilm-associated infections, one of the major threats in the field of bacterial infections [155]. Similar to the fungal biofilm structure, bacteria as well usually acquire significant resistance against conventional antibiotics and the immune system defenses, thanks to the features of the biofilm itself, including the matrix of extracellular polymeric substances produced by the same microorganisms. Of the several molecules that have been already studied for their antibiofilm activity, dendrimeric AMPs seem particularly promising, in particular by displaying the property to inhibit biofilm formation in host-mimicking conditions [156,157].

A suitable instance of both the limitations to therapeutic use inherent to the nature itself of AMPs and the ways to overcome these is offered by the recent study of Wang Manchuriga and colleagues on temporins [158]. As many natural AMPs isolated from the skin of anuran amphibians (frogs and toads), temporins display a potent antimicrobial activity but this quality is often thwarted by elevated cytotoxicity, in particular against erythrocytes [159]. Working on temporin-GHa from *Hylarana guentheri*, Manchuriga and colleagues designed several analogs of the naturally-occurring sequence, modifying the type, position and number of charged residues. Some of the derived peptides displayed a significant reduction of hemolytic activity with respect to parent peptide while retaining potent antibacterial activity, but it was not possible to reduce cytotoxicity to zero without compromising antibacterial activity, confirming that a delicate balance of charge and other physico-chemical parameters (e.g., amphipathic and extension of hydrophobic surfaces) is necessary to obtain a plausible therapeutic lead [158].

Other key criteria of AMPs that should always be studied in detail when considering these molecules for use in clinical settings are immunogenicity and pharmacodynamics/pharmacodynamics properties. Proline-rich AMPs (PrAMPs) are a class of membrane-permeable AMPs that have been identified more than 20 years ago in mammals and insects; they have an intracellular mode of action, inhibiting protein synthesis leading to a bactericidal outcome [160]. Apidaecin Api88 (18 aa) and oncocin Onc72 (19 aa)—PrAMPs based on natural peptides isolated from milkweed bug *Oncopeltus fasciatus*—were shown to be nonimmunogenic in mice, unless conjugated to protein carriers, a fact attributed to the small size of these molecules [161]. A pharmacokinetics analysis showed that Onc72 reached several organs within 10 min and that the peptide’s concentrations in blood were well above the minimal inhibitory concentrations for gram-negative key pathogens like *K. pneumoniae* [161]. More recently, the long-lasting post-antibiotic effect (PAE)—an important criterion of antimicrobial pharmacodynamics indicating the persistent growth of bacteria briefly exposed to antibiotics independently of host defense mechanisms—of several PrAMPs was tested, revealing prolonged PAEs against several strains of *E. coli*, *P. aeruginosa* and *K. pneumoniae* for all tested peptides but especially Api88, Api137, Bac7(1–60) and A3-APO [162]. “The PAEs presented here provide an additional hypothesis besides immunomodulatory effects that can explain the good in vivo efficacies of PrAMPs”, notwithstanding the fast clearance rate measured for some of these peptides, authors discussed [162], “This again highlights that MIC values determined for AMPs in vitro cannot be simply used to predict in vivo efficacies, as often assumed in the literature. Instead, MIC values should be seen as one important criterion among other parameters to be considered,” authors appropriately remarked [162].

One of the aspects that are often quoted in support of the (potential) use of AMPs in clinical practice is their low tendency to evoke antibiotic resistance. This tenet stems from the fact that AMPs generally (but not always, as specified above) hit the lipid component of the plasma membrane, a cellular component that is believed *per se* to be not easily modifiable in its basic physicochemical features by microbial targets. Although the slower emergence of resistance to AMPs with respect to conventional antibiotics is a reality, however, experience and much work have clearly shown that the reassuring thought that the complex phenomenon of resistance would not eventually thwart AMPs’ value, is somewhat naïve and misleading. In fact, the long coevolution of microorganisms and AMPs has spurred the development of several resistance mechanisms. These include sequestration by bacterial enzymes, proteolytic degradation of peptides, efflux pumps to remove AMPs from the periplasmic space, alteration of components of bacterial surface to reduce surface attachment and permeability, down-regulation by immunomodulation [163,164,165,166].

The concept of coevolution and its effect on the rise of bacterial resistance to AMPs’ action is well explained by the example of *Helicobacter pylori*. Sabine Nuding and colleagues tested the pattern of induction of gastric antimicrobial peptides by *H. pylori* as well as its susceptibility to the same peptides [167]. Researchers found that the induction of antimicrobial peptides, such as the inducible defensin HBD2 in the gastric mucosa by *H. pylori*, did not enhance the killing capacity against *H. pylori* itself. On the other hand, the expression levels of the constitutive defensin HBD1, inducible HBD3 and LL37, remained unchanged. Tested *H. Pylori* strains proved resistant to HBD1, but susceptible to the killing activities of HBD3 and LL37. “The combination of selective defensin induction and resistance to others may enable *Helicobacter* to colonize the gastric mucus layer where it can adhere to epithelial cells and induce inflammatory as well as malignant processes,” concluded the authors, that remarked the need for further studies aimed at understanding the mechanisms regarding *H. pylori* selective antimicrobial resistance [168].

Despite the limitations briefly outlined above, that have hampered their development in the classical drug discovery pipeline, AMPs are attracting continuous and ever-increasing interest as new antimicrobials agents. Out of some ~3000 molecules that have been isolated from different sources, just a handful have been the object of preclinical studies and further proceeded to clinical trials [166]. A recent analysis of AMPs patents from 2015 through 2020 has confirmed a long-standing trend, i.e., the fact that AMPs earmarked for clinical development are in vast majority analogs or derivatives of natural peptides, obtained through a template-based strategy aimed at enhancing the activity and stability of natural AMPs while reducing their toxicity [168].

Currently, just three AMPs have been approved by the U.S. Food and Drug Administration (FDA) for therapeutic use, i.e., gramicidin, colistin and daptomycin. Gramicidin has a long history. First isolated from *Bacillus brevis* over 70 years ago, gramicidin is active against a range of Gram-positive and Gram-negative bacteria, although its severe toxicity for human erythrocytes has a limited clinical indication to topical applications [169]. Polymyxin and colistin, which are cationic peptides in use for decades, have regained interest lately, due to their strong activity against multi-drug resistant Gram-negative pathogens. Their ability to bind the lipid A component of LPS makes them precious, the last resource weapons to fight septic shock, notwithstanding their known nephrotoxicity. Resistance has emerged, however, and is spreading at an alarming pace, putting the effectiveness of these valuable therapeutics at risk [170,171]. Last but not least, daptomycin. This membrane-active cyclic lipopeptide has received the green light from the FDA in 2003 to treat Gram-positive infections. It is believed that its mechanism of action differs from that of other AMPs since daptomycin causes bacterial membrane depolarization rather than membrane disruption and pore formation [172]. In recent years, resistance in *Staphylococcus aureus* has been more and more frequently reported, and the search for substitutes that might prolong the clinical use of this important antibiotic is actively underway [173].

The concern caused by AMPs resistance is clearly transmitted by a very recent clinical trial aimed at evaluating the efficacy of oral colistin-neomycin in preventing multidrug-resistant *Enterobacterales* (MDR-E) infections in solid organ transplant recipients. In the trial’s frame, a 14-day regimen of oral colistin and neomycin did not reduce MDR-E infections, and four liver-recipients developed colistin resistance [174]. A study of the molecular mechanisms of colistin resistance in environmental isolates of *Acinetobacter baumannii*, recovered from hospital wastewater and wastewater treatment plant, has shown that all isolates had increased levels of eptA mRNA and decreased levels of lpxA and lpxD mRNA; the eptA gene, in particular, could indicate its main role in colistin resistance through lipid A modification [175]. Authors hypothesized that when untreated hospital wastewater is released into the urban sewage, it might contain colistin-susceptible *A. baumannii*, and that resistance might emerge in wastewater itself following exposure to pollutants, such as cationic surfactants, and subsequently spread in the environment [175]. Looking at the bright side, things can always improve. Recent work has shown that kynomycin, a new daptomycin analog, was endowed with enhanced activity against both methicillin-resistant *S. aureus* and vancomycin-resistant *Enterococcus*, with improved pharmacokinetics and lower cytotoxicity than daptomycin [176]. Freshly acquired data suggest that physicochemical features like Ca^2+^ binding and Ca^2+^-mediated oligomerization could explain kynomycin’s enhanced antibacterial activity [177].

Even a hasty glance at the AMPs pipeline conveys the level of difficulty at bringing these molecules to the market, either for topical or systemic treatment [166,178]. After many failures, however, a couple of promising candidates loom on the horizon, at least for some therapeutic indications. Polyphor is developing the synthetic lipopeptide murepavadin, a member of a novel class of antibiotics that combine high-affinity binding to both LPS and outer membrane proteins, resulting in high specificity towards Gram-negative bacteria and effective bactericidal activity. Murepavadin, in particular, targets the lipopolysaccharide transport protein D (LptD), an outer membrane protein on *Pseudomonas aeruginosa*, leading to cell death. Phase 3 clinical trials investigating the safety and efficacy of intravenous murepavadin have been prematurely stopped due to a rise of creatinine concentration in the serum of patients treated with the AMP, indicating renal failures [179,180]. Despite these disappointing results, Polyphor plans to continue the development of inhaled murepavadin to treat chronic *P. aeruginosa* infections associated with cystic fibrosis. Exeporfinium chloride (XF-73), a derivative of AMP concept containing two cationic ammoniums and one porphyrin core, is currently the main protagonist of the anti-infectives program at Destiny Pharma [181]. XF-73 is a membrane-active antibiotic, particularly potent against Gram-positive bacteria, including MRSA. A phase 2 trial of XF-73 for the prevention of post-surgical staphylococcal nasal infections is ongoing. An in vitro study of bacterial resistance that compared XF-73 to standard antibiotics currently in use did not demonstrate the emergence of any resistance to XF-73 even after 55 repeat exposures [182].

## 7. Conclusions

The challenging research for new antimicrobial entities is still ongoing but not without difficulties. New species of bacteria, fungi and viruses are emerging, and the most alarming fact is their intrinsic and sometimes multi-drug resistance to first-line drugs. These aspects together with the fast and global spread of resistance through horizontal transfer represent a serious threat for global health. An innovative approach involves the use of compounds inspired by nature and subsequently optimized to reach suitable features, i.e., low toxicity and strong activity. The result of this process is represented by synthetic peptides. Their broad mechanisms of action and the unlikely resistance that they generate, are important advantages and perhaps the key point for a shift towards new antimicrobial synthetic peptides-based treatments for the near future.

## Data Availability

Data are contained within the article.
